# Behavioral Avoidance - Will Physiological Insecticide Resistance Level of Insect Strains Affect Their Oviposition and Movement Responses?

**DOI:** 10.1371/journal.pone.0149994

**Published:** 2016-03-04

**Authors:** Christian Nansen, Olivier Baissac, Maria Nansen, Kevin Powis, Greg Baker

**Affiliations:** 1 Department of Entomology and Nematology, University of California Davis, Davis, California, United States of America; 2 School of Animal Biology, The UWA Institute of Agriculture, The University of Western Australia, Crawley, Perth, Western Australia, Australia; 3 Entomology Unit, South Australian Research and Development Institute – SARDI, South Australian Government, Adelaide, South Australia, Australia; French National Institute for Agricultural Research (INRA), FRANCE

## Abstract

Agricultural organisms, such as insect herbivores, provide unique opportunities for studies of adaptive evolutionary processes, including effects of insecticides on movement and oviposition behavior. In this study, *Brassica* leaves were treated with one of two non-systemic insecticides and exposed to two individual strains (referred to as single or double resistance) of diamondback moth (*Plutella xylostella*) (DBM) exhibiting physiological resistance. Behavioral responses by these two strains were compared as part of characterizing the relative effect of levels of physiological resistance on the likelihood of insects showing signs of behavioral avoidance. For each DBM strain, we used choice bioassays to quantify two possible types of behavioral avoidance: 1) females ovipositing predominantly on leaf surfaces without insecticides, and 2) larvae avoiding insecticide-treated leaf surfaces. In three-choice bioassays (leaves with no pesticide, 50% coverage with pesticide, or 100% coverage with pesticide), females from the single resistance DBM strain laid significantly more eggs on water treated leaves compared to leaves with 100% insecticide coverage (both gamma-cyhalothrin and spinetoram). Females from the double resistance DBM strain also laid significantly more eggs on water treated leaves compared to leaves with 100% gamma-cyhalothrin, while moths did not adjust their oviposition behavior in response to spinetoram. Larvae from the single resistance DBM strain showed a significant increase in mobility in response to both insecticides and avoided insecticide-treated portions of leaves when given a choice. On the other hand, DBM larvae from the double resistance strain showed a significant decrease in mobility in response to insecticides, and they did not avoid insecticide-treated portions of leaves when given a choice. Our results suggest that pest populations with physiological resistance may show behavioral avoidance, as resistant females avoided oviposition on leaves without gamma-cyhalothrin. Thus, physiological resistance and behavioral avoidance do not appear to be controlled by the same selection pressures, and the mechanisms responsible for behavioral avoidance may vary among life stages. Our analysis also suggested that a population with lesser physiological resistance to insecticides may be under a stronger selection pressure and therefore be more likely to develop avoidance behaviors than a population with higher levels of physiological resistance.

## Introduction

Agricultural cropping systems provide unique opportunities for studies of behavioral adaptations and evolutionary processes, because: 1) crop management practices impose strong selection pressures, 2) a wide range of environmental variables can be experimentally controlled, 3) replicated field conditions at considerable spatial scales are fairly easy to establish, and 4) interactions among competing species and among species on different trophic levels can be manipulated and therefore studied in detail. Regarding agricultural herbivores, another important aspect of adaptations and evolutionary processes is that organisms are represented by wide ranges of diets and feeding guilds [1, 2]. As an example of behavioral adaptation by agricultural insects, a recent study described species adaptation by the peach potato aphid (*Myzus persicae*) under agricultural selection pressures [3]. This aphid species has a wide host range, and a subspecies has recently shifted to tobacco and the authors described the molecular mechanisms associated with resistance to both a potent plant defense compound (nicotine) and to neonicotinoid insecticides. There are also important bodies of research into how selection pressures induced by pesticides, both directly and indirectly, affect beneficial insects [4] and agricultural arthropod pests [5] in terms of their spatial and temporal distributions, life history traits, and behavior. The overall outcome of such physiological and behavioral adaptations to selection pressures is a perceived “resistance” of the target species to a given pesticide.

Development of “physiological” insecticide resistance is widely documented and reviewed [1, 6, 7], and it has been defined by IRAC (Insecticide Resistance Action Committee) members as: “*a heritable change in the sensitivity of a pest population that is reflected in the repeated failure of a product to achieve the expected level of control when used according to the label recommendation for that pest species*” (http://www.irac-online.org/about/resistance/). The most common mechanisms of physiological resistance are: 1) catabolic processing of the active ingredient, 2) changes in binding sites that are targeted with a given toxin, 3) decreased uptake rate, and 4) binding of toxin to sites with no toxic effect [8, 9]. “Behavioral resistance” (behavioral avoidance) has been documented for more than 40 years [10], and it is described as: “*Resistant insects may detect or recognize a danger and avoid the toxin*. *Insects may simply stop feeding if they come across certain insecticides*, *or leave the area where spraying occurred (for instance*, *they may move to the underside of a sprayed leaf*, *move deeper in the crop canopy or fly away from the target area)*.” (http://www.irac-online.org/about/resistance/mechanisms/). A critically important aspect of the likelihood of species developing behavioral resistance is the complex of sublethal effects, including insecticidal effects on: learning, neurophysiology, longevity, immunology, fecundity, sex ratio, and behavior [4]. As an example of behavioral avoidance, control of German cockroaches [*Blatella germanica* L. (Dictyoptera: Blattellidae)] in restaurants and food warehouses were largely based on glucose-based attracticides in the late 1980’s and early 1990’s, but in many regions these are no longer effective, because the cockroaches avoid feeding on the bait [11, 12]. That is, high selection pressures have favored cockroaches avoiding the glucose-based attracticides, but a recent study has demonstrated that being glucose-averse has important fitness costs [13]. A recent review article stated that avoidance (repellency and irritability induced by insecticides) is usually neglected in studies of performance evaluations of insecticides [5].

Diamondback moth [*Plutella xylostella* L. (Lepidoptera: Plutellidae)] (DBM) is a major pest of cruciferous crops [14], and it is responsible for management costs and lost production worth between US$ 4–5 billion per annum globally [15]. Data from the Arthropod Pesticide Resistance Database (APRD, http://www.pesticideresistance.org/) show that DBM populations have developed physiological resistance to at least 82 active ingredients. DBM has been reported as being the first insect pest to develop physiological resistance to both DDT (dichlorodiphenyltrichloroethane) [16, 17] and to *Bacillus thuringiensis* (denoted Bt toxins) [18]. Furthermore, there is strong evidence of DBM populations also developing behavioral avoidance. Behavioral responses of DBM to a foliar-applied insecticide have been studied, and it has been suggested that DBM is able to develop behavioral avoidance through oviposition site selection [19]. In addition, there is strong evidence of behavioral avoidance by DBM to permethrin [20, 21].

The main objective of the present study was to quantify two possible types of behavioral avoidance: 1) under choice conditions with leaves having different levels of pesticide spray coverage (including an untreated control leaf), females oviposit predominantly on leaf surfaces without insecticides, and 2) larvae avoiding insecticide-treated leaf surfaces. As a model system, we studied movement and oviposition responses by two strains of DBM denoted “single resistance” and “double resistance” based on their levels of physiological resistance to two insecticides: gamma-cyhalothrin and spinetoram. Behavioral responses by these two strains were compared as part of characterizing the relative effect of levels of physiological resistance on the likelihood of insects showing signs of behavioral avoidance. Although we are unaware of any theoretical framework providing clear predictions of expected behavioral responses by phenotypes with different of physiological resistance, we predicted that: 1) DBM individuals with confirmed physiological resistance to a given combination of dosage and insecticide show similar movement and oviposition responses to host plant surfaces with/without insecticides, and 2) DBM individuals should avoid insecticide treated surfaces and show significant changes in movement and oviposition behavior, if they are exposed to a combination of dosage and insecticide to which they are susceptible. This study highlights a consistent association between physiological resistance and avoidance responses by ovipositing females. In addition, larvae from the single resistance strain moved significant faster than those from the double resistance strain, when the entire arena was treated with either gamma-cyhalothrin or spinetoram. Our study highlights the importance of conducting behavioral studies as part of characterizing effects of selective pressures by insecticides and as part of performance evaluations of insecticides.

## Materials and Methods

### Insects

#### Waite reference DBM strain

A susceptible laboratory population of DBM (Waite susceptible strain) has been maintained on seedling cabbage, *Brassica oleracea* L. variety capitata ‘Green Coronet’ leaves in the laboratory at 25 ± 0.5°C and a photoperiod of 14:10 (L:D) h in a separately caged laboratory culture at the Waite Campus, South Australia, without exposure to any insecticides for ~22 years (~280 generations). Individuals from this strain were only included in the resistance test as a reference population.

#### Single resistance strain

A DBM field population was collected ~50–100 larvae and/or pupa from a field crop of cabbage in the Lockyer Valley region of Queensland, Australia in October 2012. The field population was received at the Waite Campus, South Australia where it was reared in an isolated cage in a control temperature room on toxin free mature 8–10 week old cabbage plants using the same methodology mentioned above for the Waite Reference population. At the time of filed collection, this DBM strain was already showing confirmed physiological resistance to gamma-cyhalothrin.

#### Double resistance strain

A DBM field population was collected ~50–100 larvae and/or pupa from a field crop of brassicas in the Werombi region of NSW, Australia in March 2010. At the time of field collection, this DBM population presented higher than typical fitness (resistance ratio of 8.4 times higher than the Waite reference population at a LC99 value) when exposed to the commercial product Success^™^ (spinosad 240g L^-1^). It was reared in the laboratory as indicated above with the exception of being provided with cabbage plant material that had been pre-treated with sub-lethal concentrations of Success^™^ approximating an LC10 concentration (a concentration rate that would cause 10% mortality). They were selected with a treatment rate of 0.06mg L^-1^ spinosad sprayed with a pressurized hand sprayer (Hills) on plants until run-off for 30 generations followed by a selection rate of 0.6mg L^-1^ spinosad for eight generations. Dow AgroSciences changed the active ingredient for Success^™^ from spinosad to spinetoram under the new product name Success Neo^™^. The selected population was evaluated to the new product Success Neo^™^ demonstrating a resistance ratio of 8.5 and 11.1 times for the LC50 and LC99 respectively. The population was then selected at 0.6 mg L^-1^ spinetoram for a further 13 generations before being used in this study.

DBM larvae and adults from the single and double resistance strains were transferred from the Waite Campus in South Australia to the University of Western Australia, Shenton Park Research Station where choice bioassays were conducted. After transfer to the University of Western Australia, the colonies were reared on a variety of potted *Brassica* plants and the adult moths provided with a 10% sugar solution. Third-fourth instar larvae and adults were collected from the colony cages using insect forceps and each individual DBM was only used once.

Regarding all three DBM strains included in this study, no specific permissions were required for collections, and we confirm that the field sampling did not affect endangered or protected species.

### Insecticides

Experimental bioassays were conducted so that pesticide coverages on leaf materials were replicated and standardized. Leaves of canola (*Brassica napus* L.) and cabbage (*B*. *rapa* L.) were dipped into one of the two formulations of non-systemic insecticides from Dow AgroSciences: Trojan^®^ (active ingredient = gamma-cyhalothrin) and Success Neo^®^ (active ingredient = spinetoram). Gamma-cyhalothrin is a pyrethroid (Group 3A insecticide in http://www.irac-online.org/teams/mode-of-action/), which has been used extensively in Australia and elsewhere for control of DBM. Gamma-cyhalothrin kills insects by contact and ingestion by affecting sodium channels in the nervous system and cause hyper-excitation or nerve block. Gamma-cyhalothrin is not translaminar, and in Australia the recommended rate is 20 ml of Trojan^®^ per liter in a minimal application volume of 50 liter per ha (http://www.herbiguide.com.au/Labels/GACY150_56175-0309.pdf). Spinetoram is a Group 5 insecticide (http://www.irac-online.org/teams/mode-of-action/) registered for DBM management in canola, vegetable and forage brassicas in Australia. (http://www.dowagro.com/au/prod/success_neo.htm). Spinetoram kills insects by contact and ingestion by allosterically activating nAChRs and causing hyper-excitation of the nervous system (http://www.irac-online.org). Spinetoram is a translaminar insecticide, and in Australia the recommended rate is 150 ml per liter of Success Neo^®^ in a minimum application volume of 50 liter per ha (http://www.agtech.com.au/label/dow/success_neo_label.pdf). One liter insecticide formulations were prepared according to labeled rates: 1) 0.4 ml of Trojan^®^ [60 mg active ingredient L^-1^] and 2) 3 ml Success Neo^®^ [360 mg active ingredient L^-1^].

### Physiological resistance bioassay

Cabbage (*Brassica oleracea* L. variety capitata ‘Green Coronet’) leaf discs (90 mm diameter) were cut from washed leaves taken from 8-wk-old plants grown in an insect-free glasshouse. The leaf discs were embedded into setting agar (1%) in a 90-mm-diameter petri dish with the abaxial side of leaf discs facing upward. Each physiological resistance bioassay included eight serial concentrations plus a control (Milli-Q water, Millipore, Billerica, MA), with five replicated leaf disks for each concentration. The insecticide solutions were made up in Milli-Q water to specific concentrations in 100 ml volumetric flasks. A controlled deposit of the test insecticide was administered using a Potter Spray Tower (PST) (Burkard Manufacturing Company Limited). Ten third instar larvae were placed on each leaf disc, and then each petri dish was sprayed with a 4 ml aliquot of the test solution. By placing the larvae prior to pesticide applications, we ensured maximum exposure of DBM instars to pesticides. Once removed from the PST the leaf dishes were covered with plastic film that was secured with a rubber band (Super band). ~100–150 fine holes were then punched into the plastic film by using a micro needle to allow air exchange. The PST was calibrated before and after each bioassay allotment with a determined application of 3.50 ± 0.11 mg/cm^2^. The PST was triple rinsed with AR Acetone and Milli-Q water between each change in treatment. The treated petri dishes were placed into an incubator at 25 ± 0.5°C and a photoperiod of 14:10 (L:D) h, with the efficacy of the treatments assessed after 48 h.

### Preparation of leaf materials for choice bioassays

No-choice and choice avoidance bioassays with DBM adults were conducted in meshed cages (60 cm × 60 cm × 60 cm, BugDorm^®^, MegaView Science Co., Taichung, Taiwan). Chinese cabbage leaves were used in no-choice and choice bioassays with DBM adults: 1) leaf completely (100%) dipped into water (positive control), 2) distal half portion of leaf (50%) dipped into insecticide formulation, and 3) leaf completely (100%) dipped into insecticide formulation. Canola leaves were used in no-choice and choice bioassays with DBM larvae. Each leaf was divided into two zones along the midrib and the following treatments were tested: 1) the entire leaf was dipped in one of the two insecticide formulations or in water only (positive control), 2) one zone was dipped in one of the two insecticide formulations and the other zone was dipped in water only. After all insecticide treatments, both canola leaves and Chinese cabbage leaves were allowed to dry for 24 hours before being used in avoidance bioassays.

### Behavioral avoidance by DBM adults

Groups of eight recently emerged but unsexed adults were collected from the main colony and transferred into each cage in a laboratory under ambient temperature (20–23°C) and relative humidity (30–60%). In three-choice bioassays, one Chinese cabbage leaf from each of the three treatments was present in the same cage, and we conducted 15 replications for each treatment. In no-choice bioassays, each of the Chinese cabbage leaf treatments was tested individually with six replications, except for untreated control leaves, which was replicated 12 times. In no-choice bioassays involving sprayed Chinese cabbage leaves, numbers of eggs laid were recorded after 48 hours.

### Behavioral avoidance by DBM larvae

The bioassay was conducted in a laboratory under ambient temperature (20–23°C) and relative humidity (30–60%). In no-choice and choice bioassays with DBM larvae, we produced a 6 cm diameter arena on individual canola leaves with the perimeter constituting a thin line of sunscreen (Hamilton Quadblock^®^, Valeant Pharmaceuticals), which was found to effectively prevent the larvae from moving outside the arena. A total of 15 replications were conducted for each treatment combination: 1) water in both zones (control), 2) gamma-cyhalothrin in both zones, 3) spinetoram in both zones, 4) water in one zone and gamma-cyhalothrin in other, and 5) water in one zone and spinetoram in other. Each leaf was only used once, and one larva was bioassayed on each leaf. In each bioassay, one larva was carefully transferred to the center of the arena using insect forceps. Similar to previoulsy published studies [22], we used a video camera placed 30 cm above the arena and connected to a computer equipped with Ethovision XT^®^ software (Noldus Information Technology Inc., Leesburg, VA) to quantify the movement of individual larvae for 15 min. In Ethovision XT^®^, the dynamic subtraction method of detection and recording 16 samples per second was used, as it was found to be most effective at detecting the larvae. The following variables were quantified: 1) average amount of time (seconds) spent in each zone, and 2) the total distance travelled by each larva (cm). In addition, we calculated the average speed by each larva. All choice and no-choice bioassays with DBM larvae were conducted under a UV light source, and DBM larvae were gently painted with a fluorescent pink dye (Pro-Cure Bad Azz, www.fishpond.com.au).

### Statistical analysis

All data analysis was conducted using the statistical software SAS 9.2 for Windows (Cary, NC, USA). In the analysis of behavioral avoidance by ovipositing females, we used analysis of variance to examine the effect of DBM strain and insecticide on average total oviposition in three-choice and no-choice bioassays. The same analysis was used to examine effects of strain and insecticide on average velocity of DBM larvae when exposed to: 1) water only, 2) insecticide only, and 3) water versus insecticide. Chi-square test was used to examine oviposition results from choice bioassays with DBM adults, and separate analyses were conducted the four combinations of DBM strain and insecticide. In each analysis, the percentage of eggs per leaf was compared with 33%, which would be the expected for a random distribution of eggs among the three leaves. Chi-square test was also used to analyze percentages of time spent by DBM larvae in the treated zone in two-choice bioassays (water in one zone versus one of the two insecticides in the other). Moreover, we compared the number of observations with less than 50% of the time spent in insecticide treated zones with an expected frequency distribution of 50%. That is, no avoidance of insecticide treated zones would imply that about 50% of the DBM larvae should spent less than 50% of the time spent in insecticide treated zones. We conducted separate Chi-square tests for the four combinations of DBM strain and insecticide.

## Results

### Physiological resistance

**Fig 1:**
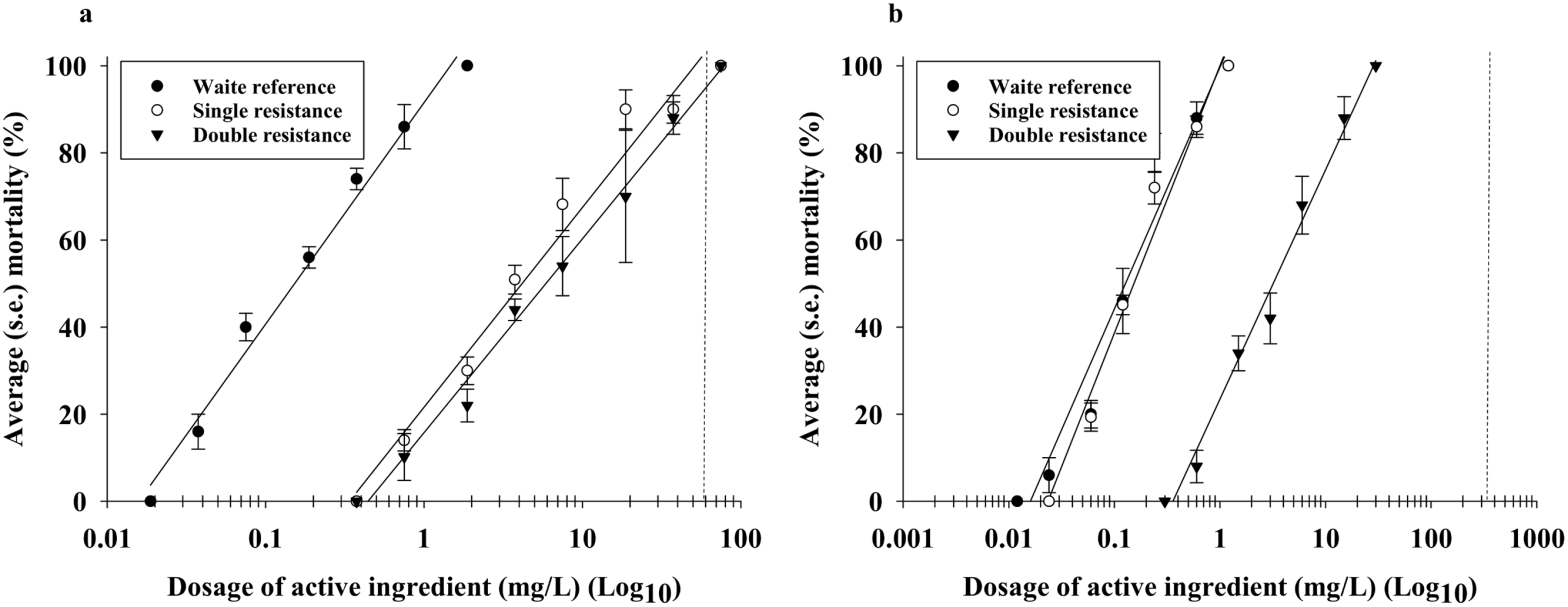
Average (s.e.) percentage mortality of third instar diamondback moth (DBM) larvae to gamma-cyhalothrin (a) and spinetoram (b). For each insecticide, we tested three DBM strains: 1) a susceptible reference strain (Waite), 2) a single resistance strain, and 3) a double resistance strain. Vertical dotted line depicts the applied rate used in bioassays.

Third instar larvae from the three DBM strains were exposed to dosages of gamma-cyhalothrin, and (Fig 1a): 1) both single and double resistance strains were about 30 times more resistant than the susceptible Waite reference strain, 2) application rate of 60 mg/L was considered high enough for third instar larvae of the Waite reference strain to be susceptible and for a small proportion of the third instar larvae of both the single-toxin and dual-toxin resistant strains to survive. From resistance bioassays with the three DBM strains exposed to spinetoram, we found that (Fig 1b): 1) resistance levels in the Waite reference and single resistance strains were very similar and about 20 times lower than in the double resistance strain, and 2) the application rate of 360 mg/L was considered high enough for all DBM third instar larvae to be susceptible to spinetoram treatments. With the single resistance strain showing high resistance to gamma-cyhalothrin and negligible resistance to spinetoram, resistance mechanisms associated with the two active ingredients were considered independent.

### Behavioral resistance by adults

**Fig 2:**
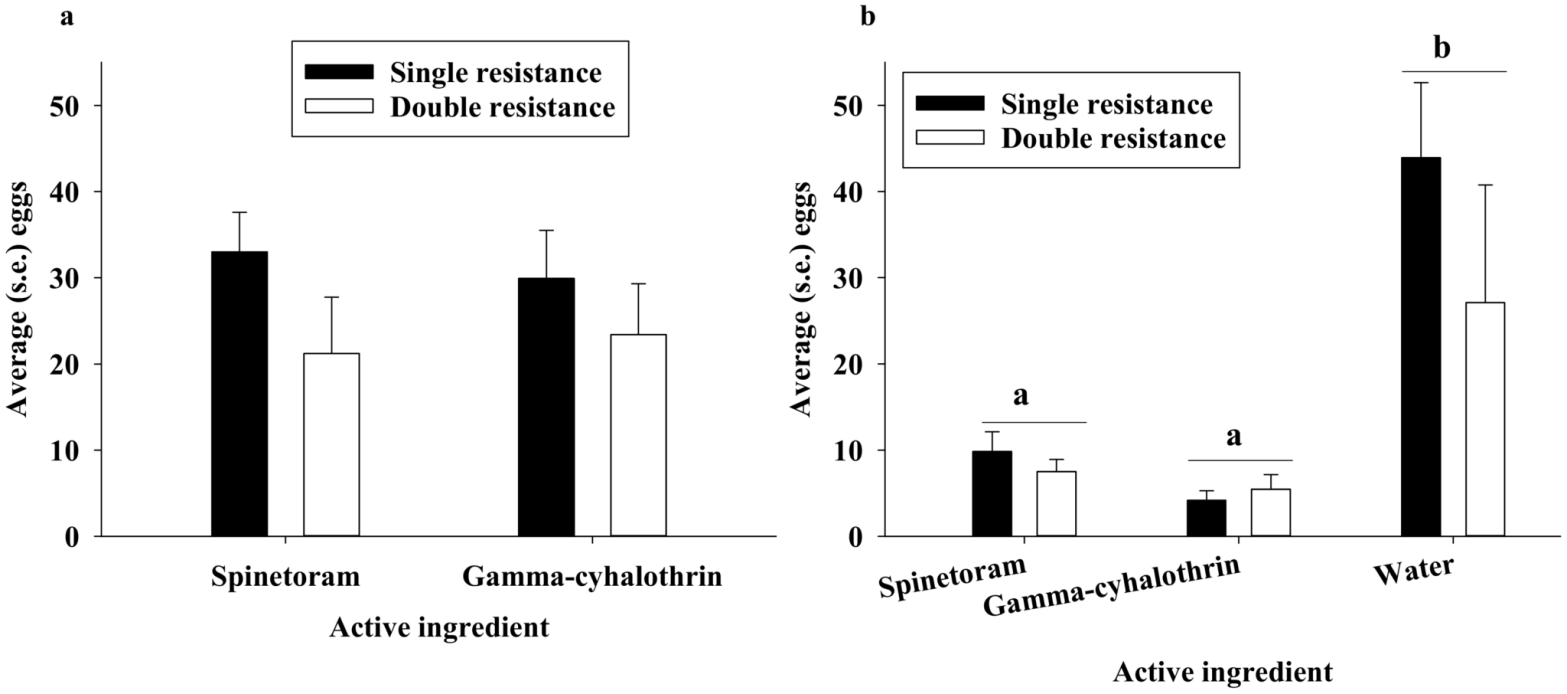
Average total oviposition after 48 hours in three-choice bioassays (a) (total sum of eggs on leaves treated with water only, and 50% or 100% insecticide treatments of spinetoram or gamma-cyhalothrin) and no-choice bioassays (b). Different letters indicate significant difference at the 0.05 level.

There were no significant effects of DBM strain (DF = 1,39, F = 1.85, P = 0.18) or active ingredient (DF = 1,39, F = 0.09, P = 0.77) on total oviposition, when DBM adults were presented with a choice of three Chinese cabbage leaves subjected to different treatments [water only (0%), distal portion of leaf (50%), and complete insecticide coverage (100%)] (Fig 2a). In no-choice bioassays with each of the three treatments bioassayed separately for the two active ingredients, there were no significant effect of DBM strain (DF = 1,72, F = 2.32, P = 0.13), but significantly more eggs were laid in bioassays with untreated leaves compared to those with treated leaves (DF = 2,72, F = 16.17, P < 0.01) (Fig 2b).

**Fig 3:**
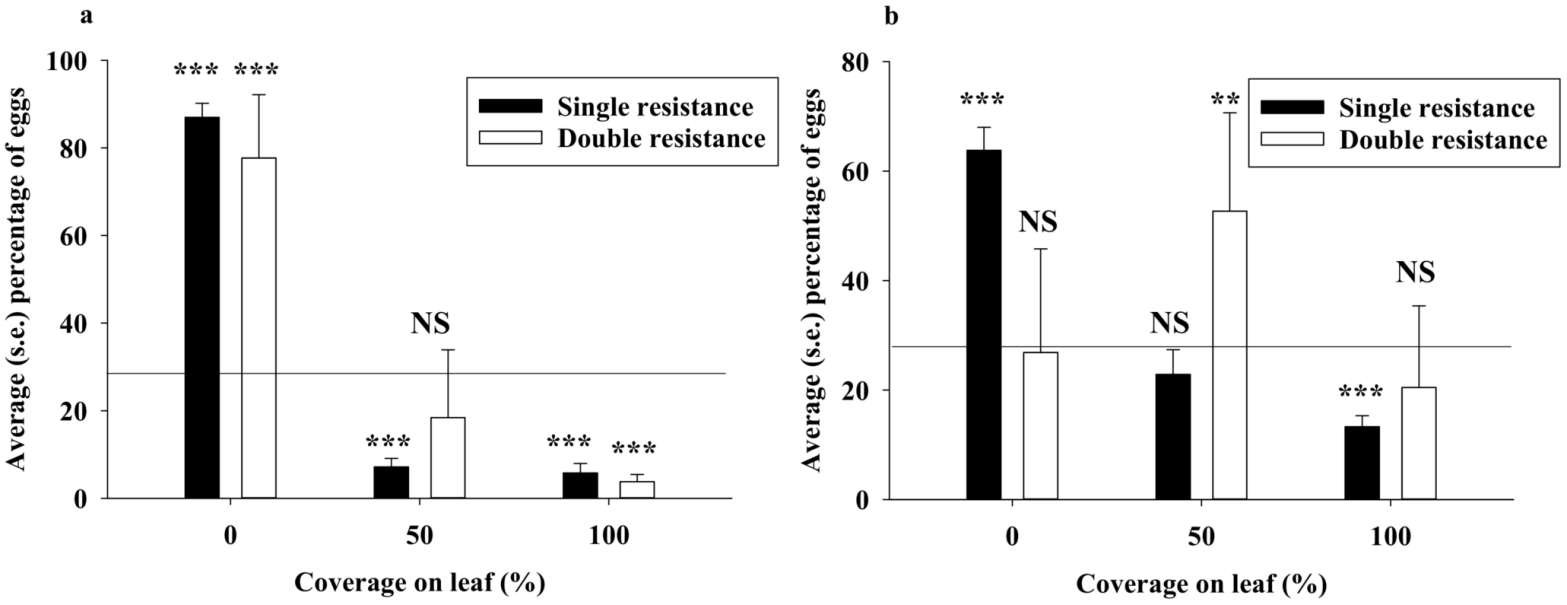
In three-choice experiment with adult diamondback moth (DBM), average percentage of eggs laid on leaves with 0% (water only), 50% coverage, and 100% coverage of gamma-cyhalothrin (a) and spinetoram (b) in bioassays with two DBM strains. Percentage of eggs laid were compared with a random distribution (33% on each leaf), which is represented by the horizontal line. “NS” denotes no significant difference at the 0.05-level, “*”denotes significant difference at the 0.05-level, “**”denotes significant difference at the 0.01-level, and “***”denotes significant difference at the 0.001-level.

In three-choice bioassays with gamma-cyhalothrin, about 80% of eggs were deposited on the leaf without insecticide, and, on average, less than 5% of the eggs were laid on leaves with 100% coverage (Fig 3a). For each combination of insecticide and DBM strain, we compared observed percentage of eggs with that of a random distribution (33%), and found that the single resistance DBM strain laid significantly more eggs than expected by random on untreated leaves, and significantly less on leaves with 50% and 100% spray coverage (Fig 3a). Regarding the double resistance strain, a similar oviposition response was observed except that the number of eggs laid on leaves with 50% gamma-cyhalothrin coverage was non-significantly different from that expected by random. In three-choice bioassays with spinetoram (Fig 3b), the single resistance DBM strain showed an ovipositional response with significantly more eggs on untreated leaves and significantly less eggs on leaves with 100% spinetoram coverage. However, the double resistance DBM strain showed a markedly different ovipositional response, the numbers of eggs laid on both untreated leaves and leaves with 100% spinetoram coverage being non-significantly different from random.

### Behavioral avoidance by larvae

**Fig 4:**
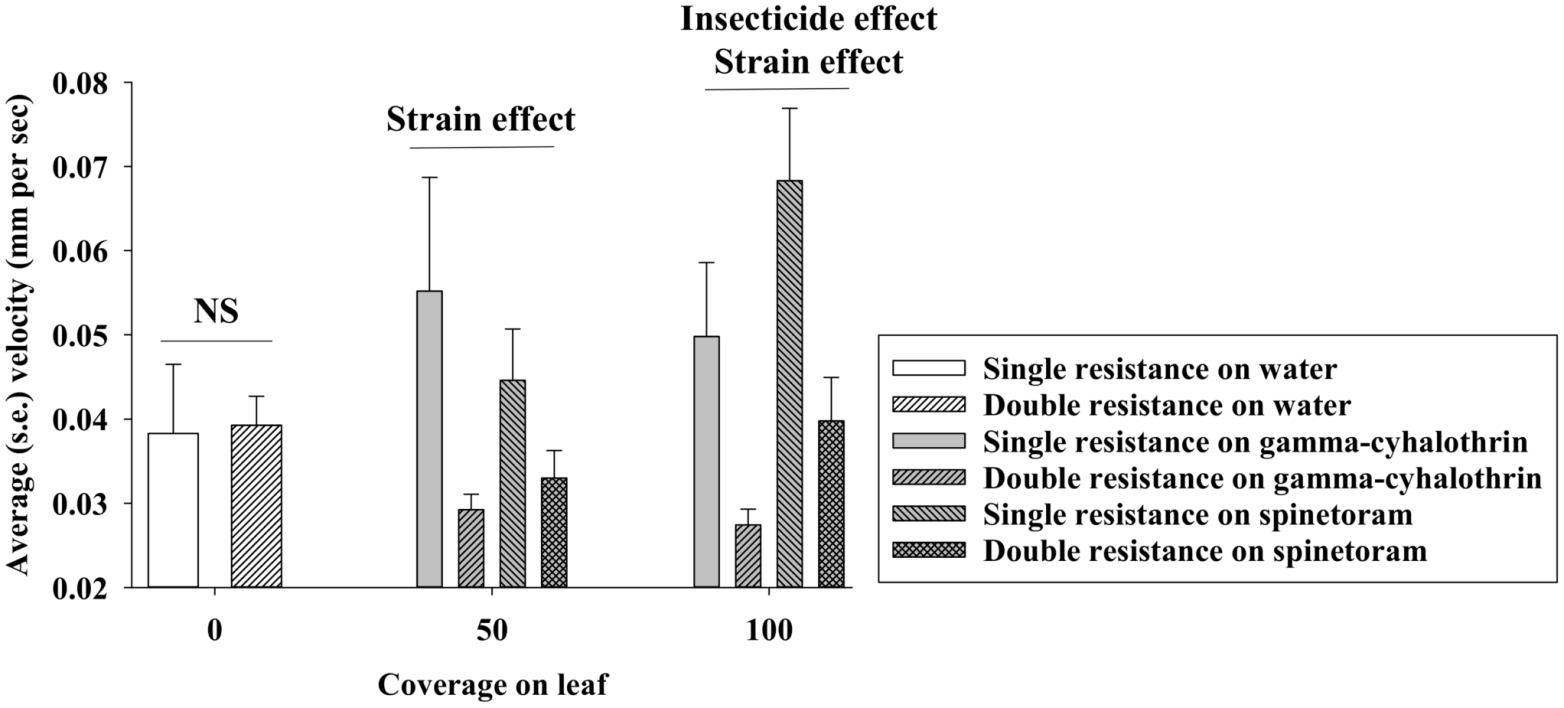
Average velocity by individual DBM larvae during 15 min bioassays on canola leaves with: 0% (water only), 50% coverage, and 100% coverage of gamma-cyhalothrin or spinetoram. “NS” denotes no significant effect of DBM strain at the 0.05-level, “Strain effect” and “insecticide effect” denote significant treatment effect at the 0.05-level.

**Fig 5:**
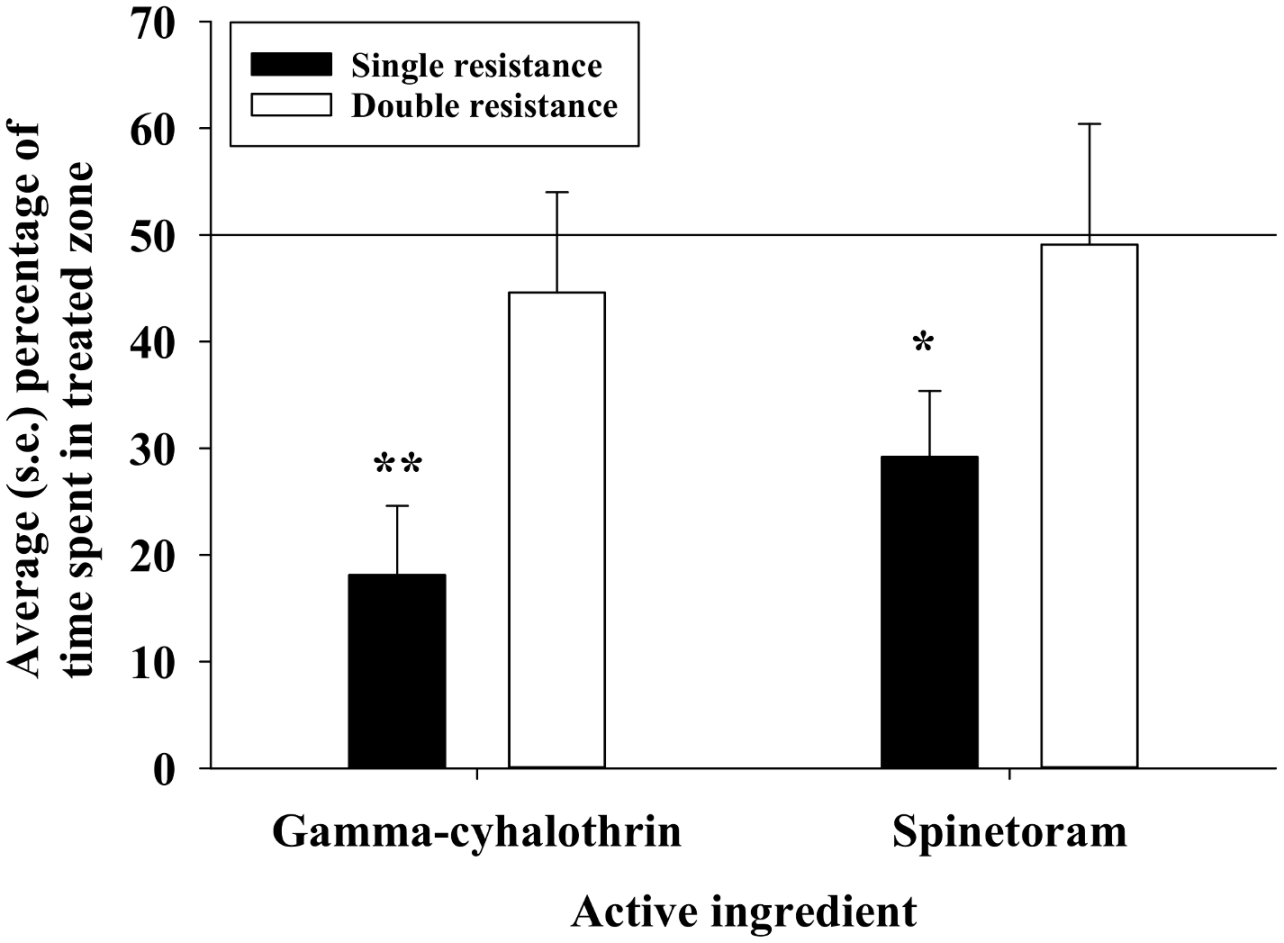
Average percentage time spent by DBM larvae in the paired treatment zones. The vertical line at 50% denotes equal time spent in treated and untreated portions of canola leaves. In comparison of DBM strains with random frequencies in treated and untreated portions of canola leaves, “*”denotes significant difference at the 0.05-level, and “**”denotes significant difference at the 0.01-level.

Average larval velocity of individual DBM larvae during the 15 min bioassays was recorded and considered an indicator of eagerness to escape from the treatment arena. With water treatment (control) in the entire arena, there was no significant difference in larval velocity between the two strains (df = 1,29, F = 0.01, P = 0.91) (Fig 4). Subsequently, we analyzed average larval velocities in bioassays with 50% insecticide coverage and found no significant effect of insecticide (df = 1,61, F = 0.11, P = 0.74) but a significant effect of DBM strain (df = 1,61, F = 6.87, P = 0.01). Regarding 100% insecticide coverage, we found significant effects of both insecticide (df = 1,59, F = 4.83, P = 0.03) and DBM strain (df = 1,59, F = 16.11, P < 0.01). For bioassays with 50% coverage, we also examined the percentage of time spent in the portion of the arena, which had been treated with insecticide, and we found that (Fig 5): 1) larvae from the single resistance strain avoided treated portions, while DBM larvae from the double resistance strain moved randomly in treated and non-treated portions of the arena.

## Discussion

DBM strains with similar levels of physiological resistance to gamma-cyhalothrin but different levels of physiological resistance to spinetoram were exposed to field rates of two insecticides, and we found that the two DBM strains: 1) Had similar total oviposition in both choice and no-choice studies. This is important, because it highlights that any sign of behavioral avoidance was not attributed to lower total oviposition. 2) Showed a high level of ovipositional avoidance in choice bioassays involving gamma-cyhalothrin. 3) Showed significantly different distributions of eggs in choice studies involving spinetoram. Thus, there was a consistent association between physiological resistance and avoidance responses by ovipositing females. Based on behavioral bioassays with DBM larvae, we found no significant difference in average velocity between strains when water was applied to leaves. However in bioassays with both insecticides and with both 50% and 100% insecticide coverage, larvae from the single resistance strain moved significantly faster than those from the double resistance strain. We also demonstrated that in bioassays with 50% insecticide coverage, only larvae from the single resistance strain avoided insecticide treated portions of leaves. The importance of including studies of sublethal effects into performance evaluations of insecticides has been acknowledged elsewhere [4, 5]. The current finding highlight the specific importance of more widespread acknowledgement of behavioral observations as part of research into pesticide performance and behavioral their effect on physiological and behavioral adaptations.

Behavioral changes by target insects exposed to insecticides may be directly induced by one or several compounds in the insecticide formulation, or they may induce an indirect innate response to minimize exposure to foreign compounds [5]. Several studies have documented behavioral avoidance in arthropod pest species. A recent study quantified the behavioral response by spider mites [*Tetranychus cinnabarinus* Boisduval (Acari: Tetranychidae)] to two miticides and quantified the potential consequences of behavioral avoidance based on simple assumptions regarding movement and level of pesticide repellency [23]. They concluded that: 1) without repellency, pesticide performance is positively correlated with target pest mobility, and 2) if the pesticide is repellent, the probability of exposure decreases, especially for a less mobile pests. Both spinetoram and gamma-cyhalothrin are labeled to kill the target pest through contact and ingestion without a clear indication of which of the two modes of action is most important. However, both modes of action require direct contact with treated host plant surfaces. There are previous reports of arthropod pests showing behavioral avoidance to pyrethroids, like gamma-cyhalothrin [6,20,24,15,16]. However, we are not aware of any published studies reporting behavioral avoidance by insects to spinetoram.

The applied rate of spinetoram was much higher than the physiological resistance level in both strains, so we predicted that both ovipositing females and larvae from both DBM strains would show avoidance to treated leaf surfaces. While ovipositing females from both strains avoided leaf surfaces treated with spinetoram, larvae from the double resistance strain did not show an avoidance response to this insecticide. One possible explanation is that a population with low physiological resistance to a wide range of insecticides may be under a stronger selection pressure and therefore are more likely to develop behavioral avoidance than a population with higher levels of physiological resistance to a wide range of insecticides.

DBM is considered one of the most economically important pests of cruciferous crops globally [15], and it is also among the arthropod pests with the highest ability to develop physiological resistance to insecticides (APRD, http://www.pesticideresistance.org/). Whalon, Mota-Sanchez (7) raised the important point that, while there is a steady increase in reported cases of pesticide resistance, the number of new arthropod species with documented resistance is not increasing nearly as fast. This suggests that a given pest species’ ability to continue to develop pesticide resistance to new pesticides is one of the key traits of economically important pests. In other words, economically important arthropod pests may share certain physiological and behavioral traits, which enable them to successfully persist in commercial/agricultural food production systems despite vast efforts by humans (including pesticide applications) to suppress their abundance and distribution. If so, it may be argued that improved insight into adaptive behavioral mechanisms and effects of anthropogenic selection pressures, such as pesticide applications, on insect species and food webs in agricultural systems is essential for the development of successful and more sustainable pest management strategies. Results from this study highlight the importance of sublethal effects of insecticides [4] with particular reference to behavioral avoidance as part of monitoring and quantifying: 1) possible shifts in population structures as a consequence of insecticide-induced selection pressures, 2) behavioral adaptations as part of evolutionary processes, and 3) effects of anthropogenic selection pressures, such as pesticide applications, on insect species and food webs in agricultural systems.
